# Evaluation of Methods for the Extraction of Microbial DNA From Vaginal Swabs Used for Microbiome Studies

**DOI:** 10.3389/fcimb.2019.00197

**Published:** 2019-06-06

**Authors:** Valentina Mattei, Selvasankar Murugesan, Muna Al Hashmi, Rebecca Mathew, Nicola James, Parul Singh, Manoj Kumar, Arun Prasath Lakshmanan, Annalisa Terranegra, Souhaila Al Khodor, Sara Tomei

**Affiliations:** Research Branch, Sidra Medicine, Doha, Qatar

**Keywords:** 16S sequencing, vaginal swabs, DNA extraction, microbiota, metagenomics

## Abstract

**Background:** The composition of the microbiome in human body sites plays an important role in health. The vaginal environment is colonized by several species of bacteria that have a major influence on reproductive health. The advancement of sequencing technologies has made the assessment of the composition of the microbiota possible through microbial DNA extraction and sequencing. Therefore, it is of a paramount importance to select a sensitive and reproducible DNA extraction method, that facilitates isolation of microbial DNA with a sufficient quantity and purity, from microbial species living in the vaginal environment. Here, we have evaluated four different DNA extraction protocols from self-collected vaginal swabs.

**Methods:** Five healthy female volunteers were enrolled in the study. Each donor was asked to self-collect 4 samples using Copan ESwab. DNA was extracted using Qiagen DNeasy kit and three modified protocols of the MoBio PowerSoil kit (“DNeasy PowerSoil” after acquisition from Qiagen). DNA quantity and integrity was checked through Nanodrop and LabChip GX. DNA was further tested through quantitative real-time PCR (qPCR) and 16S sequencing. Vaginal microbiota diversities were determined using MiSeq-Illumina high-throughput sequencing of bacterial 16S rDNA V1–V3 fingerprint. Sequencing data were analyzed using QIIME pipeline.

**Results:** Qiagen DNeasy protocol resulted in the highest DNA yield as compared to the modified protocols of MoBio Powersoil kit. The size of the DNA extracted using each protocol was ~40 kb. Qiagen DNeasy protocol gave the highest Genomic Quality Score (average ± standard deviation: 4.24 ± 0.36), followed by the different MoBio Powersoil protocols. A substantial variability in microbial DNA abundance was found across the protocols. The vaginal microbiota of the healthy volunteers was dominated by *Lactobacillus species*. MoBio Powersoil kit provided a significantly higher alpha diversity as compared to the Qiagen DNeasy kit, while beta diversity measures did not reveal any significant cluster changes, except when the Bray-Curtis method was applied.

**Conclusion:** We were able to isolate microbial DNA from the vaginal swabs. Qiagen DNeasy method gave the highest DNA yield and quality but was not optimal in detecting microbial diversity. The modified MoBio PowerSoil protocols showed higher microbial diversities as compared to the standard protocol.

## Introduction

The diverse composition of the microbiota inhabiting different human body sites plays an important role in human physiology, immunity, and nutrition (Rosenstein et al., 1996; Mazmanian et al., 2005; Ley et al., 2006a,b; Turnbaugh et al., 2006, 2007; Dethlefsen et al., 2007; Ling et al., 2010). The vaginal environment represents one of the most diverse habitats for bacteria which are considered the major players in providing antimicrobial defense mechanisms to protect women against various diseases (Sobel, 1999). In healthy reproductive-age women, the vaginal microbiome is shown to be dominated by Lactobacilli, the main producers of acidic fermentation products (such as lactic acid) to ensure an acidic vaginal environment that restricts the growth of most pathogens (Witkin et al., 2007). The human vagina and the colonizing microbes interact in a mutualistic relationship. Any imbalance in the vaginal microbiome composition, also known as dysbiosis, could lead to a disease state (Fredricks et al., 2005). Understanding the composition of the vaginal microbial ecosystem is essential to study its dynamics and comprehensively dissect the etiology of vaginal diseases and their role in women's reproductive health (Ling et al., 2010).

Not all the species that reside in the vagina can be cultivated (Rappe and Giovannoni, 2003). That has impeded the full characterization of the vaginal microbiota until the development of high throughput DNA sequencing technologies (e.g., the sequencing analysis of the small ribosomal subunit 16S), that have revolutionized the bacterial detection methods (Pavlova et al., 2002; Lamont et al., 2011). In contrast to the traditional isolation methods which are prone to biases due to differential bacterial growth ability, sequencing allows direct examination of the DNA content in a sample. Nevertheless, significant methodological challenges should be tackled to allow consistency and reproducibility of the results obtained (Sinha et al., 2015).

The characterization of different microbial communities through high throughput sequencing technologies is exposed to a number of pitfalls that influence the final outcome. Biases introduced in the sample processing steps can compromise the reliability of the sequencing results, hence they should be avoided. Sources of bias exist at each step of the experimental pipeline, and range from the sample collection methods to the DNA extraction protocols used and sequencing artifacts among others (Brooks et al., 2015). It is also worth mentioning that an efficient extraction of a high quality DNA from limited amounts of samples is the key challenge for cutting edge downstream applications like Next Generation Sequencing (NGS). Therefore, the selection of a reliable method for DNA extraction is of paramount importance to ensure a high DNA yield and a representative characterization of microbial communities in any given body site. Standardizing the methods used to ensure reliable and reproducible analysis of vaginal samples is required for pursuing studies on the vaginal microbiome.

Self-collected vaginal swabs provide privacy; they can be conveniently performed at any time according to the comfort of individual and are financially affordable for field-based longitudinal cohort studies requiring tedious repeated sampling. A comparative study between self-collection and clinician-collected vaginal swab samples showed similar microbial diversity using both techniques in US women (Forney et al., 2010). While there has been much attention and efforts pertained to the methods used to collect the vaginal samples (Forney et al., 2010; Mitra et al., 2017), there has been less work performed on assessing what extraction methods should be used in order to maximize the release of microbial DNA carried in the fibers of the swabs and to reflect an accurate image of the complexity of the microbial communities.

The aim of this study was therefore to compare four methods for the extraction of microbial DNA from self-collected vaginal swabs used for microbiome studies. We have tested the standard Qiagen DNeasy Blood and Tissue DNA extraction kit and three modifications of the MoBio PowerSoil protocol (currently “DNeasy PowerSoil” after acquisition of MoBio by Qiagen in 2016; Carlsbad, CA). DNA extracted with those methods was checked for quantity and quality and further assessed through qPCR and 16S sequencing on Illumina MiSeq.

## Materials and Methods

### Sample Collection

Five adult female non-pregnant donors were enrolled in this study, after they signed an informed consent, approved by Sidra Institutional Review Board committee (IRB protocol #1709014294). The donors were self-reported as healthy. All methods were performed in accordance with the relevant guidelines and regulations. Each donor was asked to collect 4 vaginal samples using Copan ESwab (Copan Diagnostic Inc., Murrieta, CA, USA). The Copan ESwab is a collection and transportation system which incorporates a modified Liquid Amies transport medium and a flocked swab. The samples were self-collected by the volunteers as follows: using the non-dominant hand to open the labia, the swab is inserted into the vagina, then twisted several times while inside of the vagina. Once the swab was collected, it was placed immediately into the ESwab transport tube containing transport medium. Swabs were then transported directly to the laboratory (within 2 h) and stored at −80C before processing.

### Microbial DNA Extraction Methods

To extract microbial DNA from all vaginal samples collected, four different DNA extraction protocols were tested and evaluated:

1. Qiagen DNeasy Blood and Tissue (Qiagen, Venlo Netherlands) referred to as method #1 hereafter.
2. MoBio PowerSoil with C2 and C3 solutions combined (method #2).
3. MoBio PowerSoil with C1, C2 and C3 solutions combined (method #3).
4. MoBio PowerSoil standard protocol (method #4).

**Figure 1:**
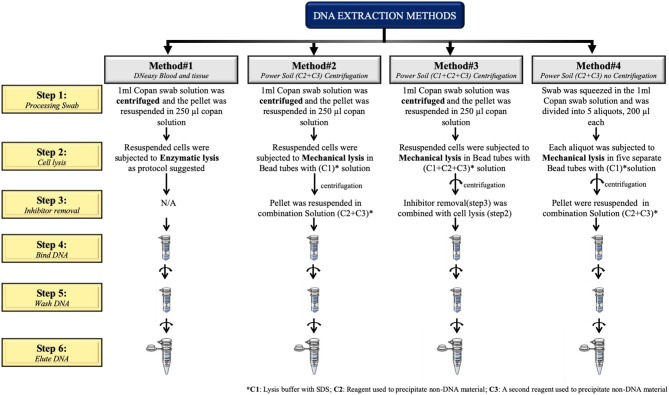
Schematic representation of the four extraction protocols tested in this study.

The first three protocols (methods #1–3) use a pre-centrifugation step (10 min at 7,500 rpm) in order to collect the pellet prior to cell lysis. All protocols are described in Figure 1.

### DNA Quality Control

The assessment of DNA quantity and quality was carried out using Nanodrop. The absorbance at 260 and 280 nm wavelengths was measured. Most of the isolated DNA samples had an OD260/OD280 ratio between 1.72 and 2.35. We then checked the DNA integrity using LabChip GX (PerkinElmer, Waltham, Massachusetts, United States). The Gel-Dye solution, DNA samples and DNA ladder were prepared according to the manufacturer's instructions; the run data was compared to the electropherogram of a typical high molecular weight ladder and assessed for quality. A genomic DNA (gDNA) quality score (GQS) was calculated for each sample. The GQS is derived from the size distribution of the gDNA and it represents the degree of degradation of a given sample, with a score of 5 corresponding to intact gDNA and a score of 0 corresponding to a highly degraded gDNA.

### Quantitative Real Time PCR (qPCR)

To evaluate the relative abundance of bacteria as compared to human DNA in samples tested, a quantitative real time PCR (qPCR) was performed to assess the presence and the relative quantity of microbial DNA. Universal bacterial primers amplifying the V3 region of 16S rDNA gene (341 F and 534 R) were used to amplify approximately 194 bp as described previously (Whiteley et al., 2012). Primers for the Firmicutes family were used as representative for the vaginal microbial DNA (Yang et al., 2015). STAT2 (RefSeq: NC_000012.12) primers were used as representative for the human DNA. The primers used are detailed in Table 1.

**Table 1:** List of primers used for qPCR.

	**Forward**	**Reverse**
V3 of 16S rDNA gene	5'-CCTACGGGAGGCAGCAG-3′	5′-ATTACCGCGGCTGCTGG-3′
Firmicutes	5′-GGAGYATGTGGTTTAATTCGAAGCA-3′	5′-AGCTGACGACAACCATGCAC-3′
STAT2	5′-TGAGGCCTTCAGGAAGTTGG-3′	5′-CCACATTTGTTCCCGTCTCC-3′

Each PCR reaction was performed in a 20 ul final volume containing 200 nM of forward and reverse primers, 10 μL of 2X KAPA Master Mix (Sigma-Aldrich, St. Louis, WI, USA). Input DNA was 50–100 ng/reaction. Cycling conditions were as following: initial denaturation at 95°C for 10 min; 30 cycles at 95°C for 30 s, 63°C for 30 s, and 72°C for 30 s; final step at 72°C for 10 min and hold at 4°C. Reactions were run on the LightCycler 480 System (Roche, Basel, Switzerland). To verify primers specificities, melting curves were generated at the end of each PCR reaction. Fluorescent data was acquired during the extension phase. After 30 cycles, a melting curve for each gene was generated by increasing the temperature from 60 to 95°C (1°C for each step), while the fluorescence was measured. Samples were run in triplicates. For each experiment a no-template reaction was included as a negative control. Two human DNA samples were used as controls. The qPCR data was analyzed using the 2^∧^-(delta Ct) method (comparing Ct of genes of interest against STAT2).

### 16S rDNA Sequencing

The V1-V3 regions of the 16S rDNA were amplified using various forward primers: 27F with 12 bp golay barcodes containing a specific Illumina 5′ adapter for each sample and a common reverse primer 534 R (Zheng et al., 2015). In brief, PCR was performed in triplicate in a 50 μL reaction mixture containing 10 ng of template DNA and 2x KAPA HiFi HotStart Ready Mix. The following thermal cycling conditions will be used: 5 min of initial denaturation at 94°C; 30 cycles of denaturation at 94°C for 30 s, annealing at 62°C for 30 s, and elongation at 72°C for 30 s; and a last step at 72°C for 10 min. The amplified PCR products of ~650 bp in size from each sample were pooled in equimolar concentrations. This pooled PCR product was purified using the FlashGel™ DNA System (LONZA). High throughput sequencing was performed on an Illumina MiSeq 2 × 300 platform (Illumina, Inc. San Diego) in accordance with manufacturer's instructions. Image analysis and base calling were carried out directly on the MiSeq.

### Sequencing Analysis for Microbial Diversity

Demultiplexed sequencing data were analyzed using QIIME software v1.9.0 pipeline (Caporaso et al., 2010). FASTQ files were converted into FASTA files, and all demultiplexed files were concatenated into a single file. Further analysis was performed as previously reported (Murugesan et al., 2015; Garcia-Mena et al., 2016). Sequence alignments were done against the Greengenes core set (Desantis et al., 2006). Alpha diversity was measured by R software, using the phyloseq package (Mcmurdie and Holmes, 2013). Beta diversity was represented using weight UniFrac distance measure (Lozupone et al., 2007) and contributions to the differences in the beta diversity were presented as principle coordinate analysis as prposed in QIIME.

### Statistical Analysis

Data were analyzed using Medcalc 11 (Medcalc Software, Stata Software). Paired Student's *t*-tests were used to compare means. Alpha diversity measures such as Observed, Chao1, Shannon and Simpson indices were calculated using minitab17 (Minitab statistical software). Kruskal wallis tests were used to compare the statistical difference among diversities between the described DNA extraction protocols. *P* < 0.05 were considered statistically significant. We applied the Linear Discriminative Analysis (LDA) Effect Size (LEfSe) tool which uses Kruskal-Wallis and estimates the effect size of the comparisons to identify the significant shift in profile to distinguish the difference in extraction methods (Segata et al., 2011). Adonis and Distance based redundancy (db-RDA) method used to calculate distance matrix difference between the extraction methods included in this study using beta diversity parameters (Sobel, 1997).

## Results

### Assessment of the Quality and Quantity of the DNA Extracted From the Vaginal Swabs

**Figure 2:**
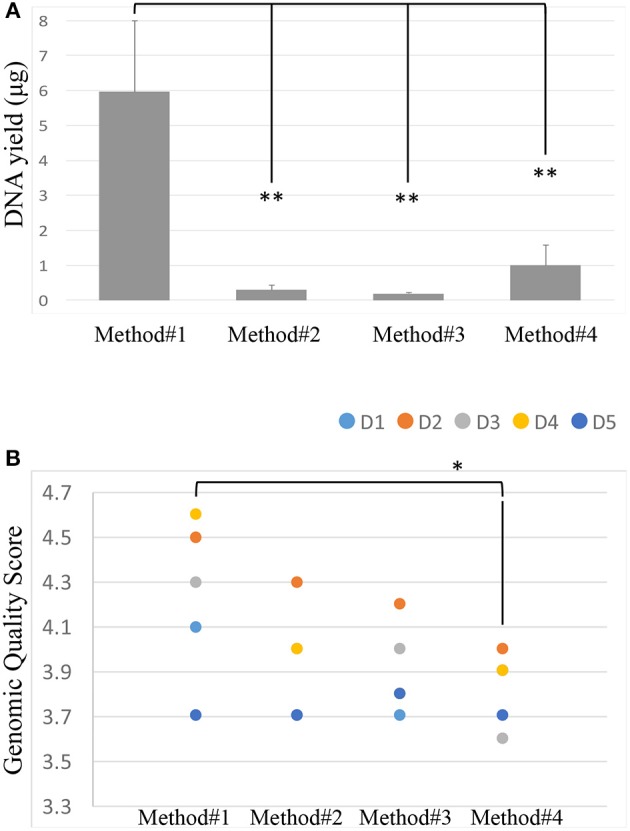
DNA yield (ug) across the 4 extraction methods. Student's *t*-test was used to compare means, **P < 0.01 **(A)**. Genomic Quality Score across the 4 extraction methods, donors are represented by colored spots. Student's *t*-test was used to compare means, *P < 0.05 **(B)**.

After isolation of the DNA from the vaginal swabs, the quality and quantity of the DNA extracted were evaluated using Nanodrop. Most of the isolated DNA samples had a similar OD260/OD280 absorbance ratio of approximately 2 (Supplementary Figure 1). We further confirmed the quality of the DNA isolated by running all the samples on the LabChip and by assessing their GQS (a representative image of GQS scoring is reported in Supplementary Figure 2). As described in Figure 2A, significant differences in the DNA yield were observed when we compared the four extraction protocols; method #1 resulted in the highest DNA yield with an average of 5.96 ug, followed by methods # 4, 2, 3, respectively.

The size of the DNA extracted was ~40 kb. The GQS was calculated for each sample and plotted in Figure 2B. Method #1 resulted in the highest GQS, with an average of 4.24, followed by methods #2, 3, 4, respectively (Figure 2B).

### Assessment of Microbial Representation in Extracted DNA by qPCR

**Figure 3:**
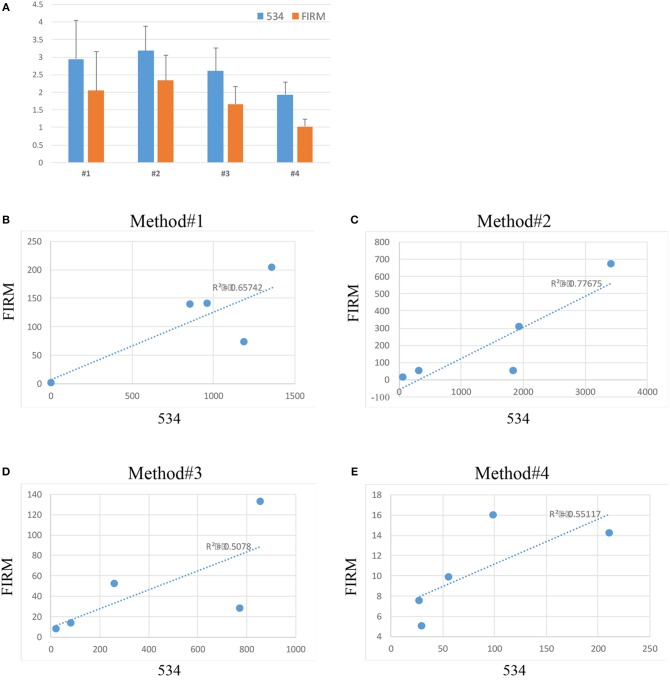
Relative abundance (2^∧^-delta Ct values on logarithmic scale) of bacteria across the 4 extraction methods (534 indicates total bacterial a abundance, FIRM indicates the abundance of bacteria belonging to the Firmicutes phylum) **(A)**. Correlation between 534 and Firmicutes microbial abundance in method #1 **(B)**, method #2 **(C)**, method #3 **(D)**, and method #4 **(E)**.

The abundance of bacterial DNA was assessed by qPCR using universal primers for the bacterial 16S rDNA gene (341 F and 534 R) and primers for the Firmicutes family which represents one of the most predominant bacteria identified in the vaginal environment in healthy women of reproductive age (Ling et al., 2010). Data was calculated with the 2^∧^-delta Ct method and plotted in a logarithmic scale (Figure 3).

Substantial variability in microbial DNA abundance was found across all the protocols tested. Overall, bacterial DNA was detected in all the extraction protocols tested, with some variabilities (Figure 3A). Method #2 gave the highest yield of bacterial abundance followed by methods #1, 3 and 4, respectively (Figure 3A). A positive correlation of Firmicutes and 16S rDNA gene abundances across all the 4 protocols was observed (Figures 3B–E), suggesting that indeed bacteria of Firmicutes family represent a good portion of the vagina flora.

### Microbiota Content Complexity and Composition

**Figure 4:**
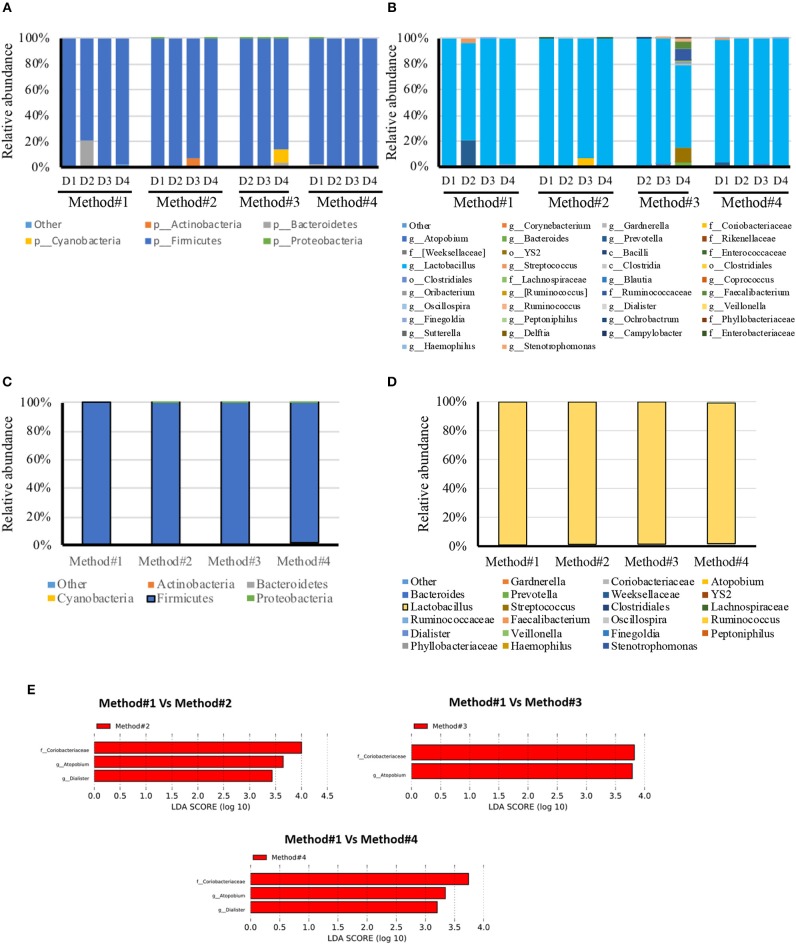
Relative abundance of bacteria in vaginal swabs of using four different DNA extraction methods. Y-axis shows % of relative abundance; X-axis indicates the abundance for each donor with four extraction methods as mentioned in Methods; each taxonomic category is shown by a different color; **(A)** phylum level for each donor included in the study, **(B)** genus level for each donor included in the study, **(C)** phylum level for each DNA extraction method included in the study, **(D)** Genus level for each DNA extraction method included in the study, **(E)** Graphs of linear discriminant analysis (LDA) scores for **(A)** Differentially abundant bacterial genera and families; among the different DNA extraction methods. LDA scores indicate overrepresented data in Method#2, Method#3, and Method#4 in comparison to Method#1 (red). Features with LDA scores≥2 are presented.

All the DNA samples isolated from the four DNA extraction methods were processed for sequencing using Illumina Miseq platform after amplifying the V1-V3 regions of the 16S rDNA gene. Clustering and annotation of the operational taxonomic units (OTUs) was performed using the same pipeline for all the samples. Average number of sequences count per sample was also calculated (Supplementary Figure 3) and a significant difference was observed when comparing extraction method #1 and #2. No statistical significance was observed when we compared other extraction methods used. A comparison of OTUs from phylum to genus levels was performed. No statistical significance was observed in the various extraction methods used when we compared the total OTUs count (Supplementary Figure 3). However, our analysis revealed that regardless of the extraction protocol used, Firmicutes remains as expected the most abundant phylum with an average of 98% of all OTUs (Figure 4C). At the genus level, Lactobacillus species was the most abundant genera (Figure 4D). Prevotella spp., Streptococcus spp., Ruminococcaceae and Coriobacteraceae were the least abundant genera across the four DNA extraction methods (Figures 4A–D). Relative abundance of the vaginal microbiota profiles of each donor using different extraction methods showed that there is a shift observed in method #2, method #3, and method #4 as compared to method #1 (Figures 4A–D). Overall, Powersoil DNA extraction methods exposed more genera especially with method #3 and method #4 as compared to method #1 (Figure 4B).

LEfSe analysis revealed the significant differences in the abundances of *Coriobacteriaceae, Atopobium* spp., and *Dialister* spp., in method #2 and #4 when compared with method #1. Likewise, Method #3 showed significant difference in the abundance of *Coriobacteriaceae* and *Atopobium* spp., in comparison with method #1 (Figure 4E).

### Microbial Diversity

**Figure 5:**
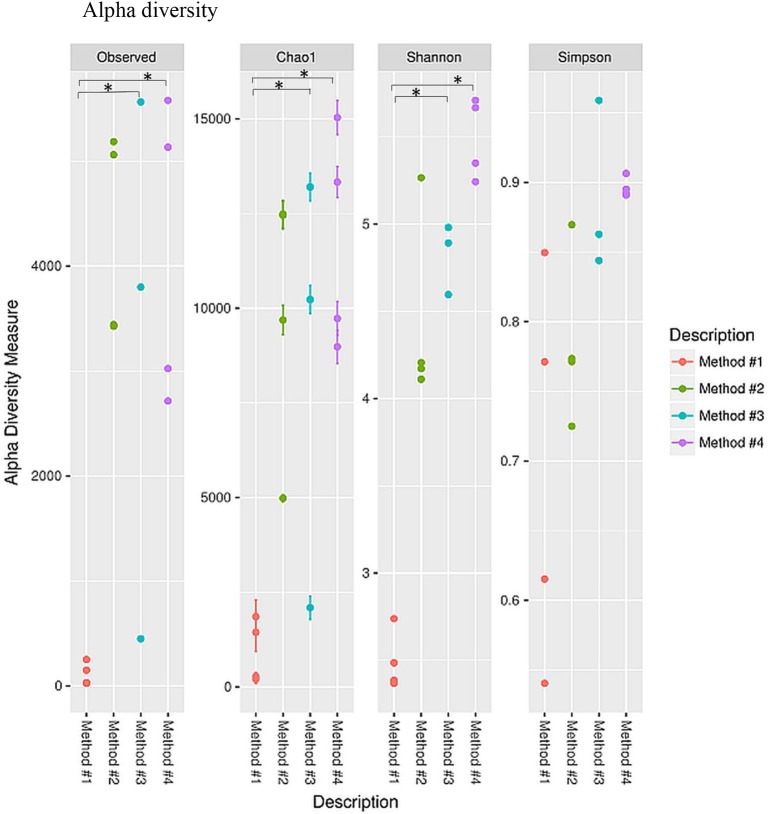
Alpha diversity measures for the vaginal microbial community of four different DNA extraction methods. Alpha diversity was measured by the number of OTUs observed or by the Chao1, Shannon and Simpson diversity measures, *P < 0.05.

Diversity indices were calculated to describe the complexity of samples (alpha) and to differentiate between the samples assessed (beta). Alpha diversity was measured using four indices (Figure 5). Alpha diversity measures (Figure 5) indicated that significant increase was observed in both chao1 (*P* = 0.021, *P* < 0.05) and observed species richness (*P* = 0.021, *P* < 0.05) in the vaginal microbial community structure when method #4 was used, and significantly higher chao1 (*P* = 0.034, *P* < 0.05) and observed species richness (*P* = 0.034, *P* < 0.05) in method #3 as compared to method #1. This result is also concordant with the Shannon diversity index in method #3 (*P* = 0.021, *P* < 0.05) and in method #4 (*P* = 0.034, *P* < 0.05) (Figure 5).

**Figure 6:**
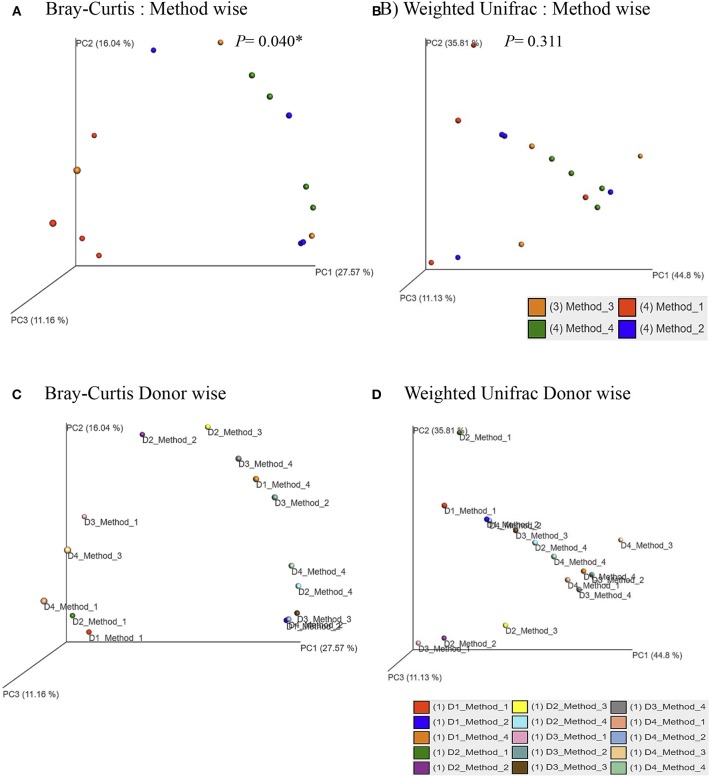
Principle Coordinates Analysis plot. **(A)** Principle Coordinates Analysis (PCoA) based on Bray–Curtis dissimilarities of microbial communities among different DNA extraction methods included in the study; **(B)** Principle Coordinates Analysis (PCoA) based on Weighted UniFrac measures dissimilarities of microbial communities found among different DNA extraction methods as mentioned in Methods; **(C)** Principle Coordinates Analysis (PCoA) based on Bray–Curtis dissimilarities of microbial communities for each donors with its respective extraction method used in the study; **(D)** Principle Coordinates Analysis (PCoA) based on Weighted UniFrac measures dissimilarities of microbial communities for each donors with its respective extraction method used in the study. Axes are scaled to the amount of variation explained; *P < 0.05.

**Figure 7:**
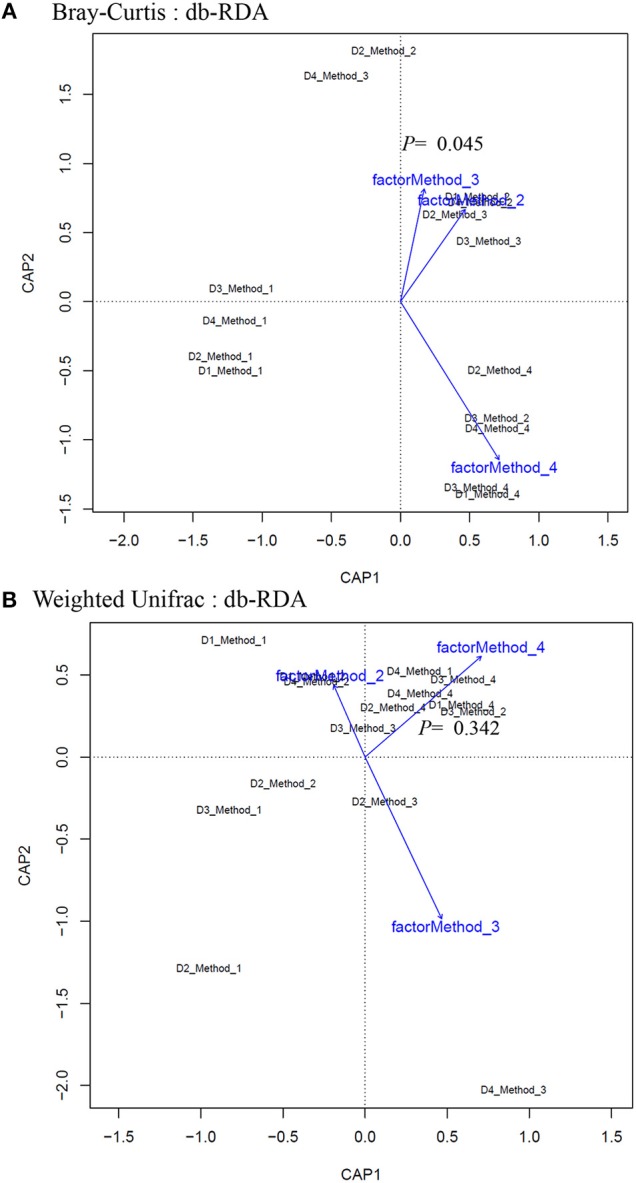
Distance-based redundancy analysis (dbRDA) plots of **(A)** Bray–Curtis dissimilarities of microbial communities among different DNA extraction methods included in the study **(B)** Weighted UniFrac measures dissimilarities of microbial communities found among different DNA extraction methods as mentioned in Methods. The dbRDA visualizes the distance-based linear model which associates the microbiota with DNA extraction methods.

Principle coordinate analysis (PCoA) of beta diversity was performed to examine whether each method clustered in a distinct pattern using both the taxonomic Bray-Curtis dissimilarities (Figure 6A) and phylogenetic weighted UniFrac distances (Figure 6B). The Adonis method was applied to calculate the variation of distances among different extraction methods. The Bray-Curtis method showed that method #1 was significantly different from other methods (Figure 6A). No significant clustering was observed when we compared the four DNA extraction methods using weighted UniFrac distances (Figure 6B). The PCoA of Bray-Curtis dissimilarities (Figure 6C) and phylogenetic weighted UniFrac distances (Figure 6D) of each donor also showed that there was no donor specific clustering irrespective of methods of extraction. A distance based redundancy analytic method was applied to calculate the variation of distances among different extraction methods. The Bray-Curtis method showed that method #1 was significantly different from other methods (Figure 7A). No significant clustering was observed when we compared the four DNA extraction methods using weighted UniFrac distances (Figure 7B).

## Discussion

The use of cost-effective sequencing methodologies for the analysis of microbial communities requires efficient and reproducible strategies for DNA extraction. Strategies used so far comprise multiple technical variables that may potentially affect the downstream applications. These steps range from sample collection method, sample collection tube, sample storage, DNA extraction and the sequencing platform used to assess the microbiome composition in any given body site. Therefore, it is vital to use a universal method at each step in order to facilitate comparison of the results generated from various studies in the same research area. A recent study (Panek et al., 2018) summarized the challenges in the methods used to study the microbiome in stool samples, however similar studies on the challenges faced when assessing the vaginal microbiome are still lacking, while timely needed.

It is well known that the vaginal environment is colonized by diverse bacteria (Ravel et al., 2011; Ma et al., 2012; Jespers et al., 2017). The majority of these indigenous microbiota exist in a mutualistic relationship with their human host and prevent the colonization of potentially pathogenic organisms, including those responsible for bacterial vaginosis, yeast infections and urinary tract infections (Hillier et al., 1992; Sobel, 1997, 1999; Gupta et al., 1998). Recent development in the next generation sequencing technologies revealed that vaginal microbiome vary among healthy women (90–100%) with genus *Lactobacillus* spp., as the predominant member to almost zero *Lactobacillus* in women with bacterial vaginosis (Xiao et al., 2016). Collection methods of the vaginal samples play an important role in reflecting the microbial composition of the vaginal ecosystem. A comparison of vaginal swab and cytobrush to collect samples to explore the vaginal microbiota composition revealed that there was no significant change neither in the species richness nor in diversity (Mitra et al., 2017). Moreover, a comparative study between self-collection and clinician collected vaginal swab samples showed similar microbial diversity using both techniques in US women (Forney et al., 2010). Although the importance of vaginal microenvironment in relation to health and diseases has been well established, there is a paucity of data regarding DNA extraction methods and their ability to assure microbial diversity in not well-characterized sites like gut, saliva and skin (Vesty et al., 2017; Lim et al., 2018; Stinson et al., 2018).

In this study, we have evaluated four different DNA extraction methods and assessed the DNA yield and quality, and the diversity of bacterial DNA extracted from self-collected vaginal swabs. The hands-on time was comparable across the protocols and all the kits evaluated extracted bacterial DNA successfully, although with some variabilities. Among the 4 methods tested, method #1 was the most efficient with respect to the amount and quality of DNA, although the other three methods resulted in a good quality DNA as well. DNA yield was significantly higher in method #1 compared to the other methods. However, method #1 showed the lowest efficiency in detecting microbial diversity as compared to the other protocols as observed in the alpha diversity measures. We cannot exclude that this may be due to inefficient enzymatic bacterial lysis and/or to the presence of host DNA along with microbial DNA. In this study, we show that DNA extraction protocol using various lysis methods may affect the vaginal microbiome composition. Method #3 was the most efficient in detecting bacterial diversity although the yield was significantly lower compared to method #1. The ultimate aim of most of the microbial community based studies is the representation of microbial diversity, which is not generally considered as a standard to evaluate DNA extraction methods (Yuan et al., 2012). Compared to the other methods, the in-house modified method #3 is the most efficient in detecting microbial diversity probably due to the ability of the combined C1,C2, and C3 solutions and mechanical bead beating to break down the bacterial cell wall of vaginal microbiota. Gram positive cell walls of members of *Coriobacteriaceae* family such as *Atopobium* spp. are thicker and more resistant to enzymatic degradation. Method #3 uses a combination of enzymatic and mechanical lysis which improves this method's efficiency in recovering both gram positive and gram negative members of the vaginal flora. Although Method #4 has edged slightly in alpha diversity, method #3 yielded DNA of higher quality when compared to method #4 (Table 2, Figure 2). As opposed to method #4, method #3 combines C1, C2, and C3 buffers in one step, which resulted in a higher efficiency in detecting different members of the vaginal microbiome environment. Additionally, method #3 has the advantage of a lower processing time compared to method #4. Method #1 clustered apart from the other methods involving mechanical lysis (Figure 6A). Obviosuly the choice of the most appropriate method for DNA extraction is based on the researcher's needs and the downstream application that will be used. The ideal DNA extraction method may depend on the specific study goals; in some instances, efficient capture of bacterial diversity within a microbial community may be more important than DNA yield (Table 2).

**Table 2:** Summary of the features of DNA extraction methods used in this study.

**Outcomes**	**Method #1**	**Method #2**	**Method #3**	**Method #4**
DNA yield	+++	+	+	+
DNA quality	+++	++	++	+
Microbial sequence count	+	+++	++	++
Microbial diversity	+	++	+++	+++

*+++, denotes high, ++, Moderate, +, Low*.

In conclusion, our results show here that despite the fact that the Qiagen DNeasy Method #1 was the most efficient kit in terms of DNA quantity and quality, the MoBio Powersoil modified Method #3 was the most efficient in capturing microbial richness and diversity.

Additional studies with a bigger cohort are warranted to confirm these findings.

## Ethics Statement

Five adult female non-pregnant donors were enrolled in this study, after they signed an informed consent, approved by Sidra Institutional Review Board committee (IRB protocol #1709014294). All methods were performed in accordance with the relevant guidelines and regulations.

## Consent for Publication

All authors have reviewed the final version of the manuscript and approved it for publication. The contents of the paper have not been published previously.

## Author Contributions

ST and SA conceived the study, wrote the manuscript, performed statistical analyses, and generated graphs. VM and MA performed the DNA isolation, DNA QC and qPCR experiments. SM, PS, and AL performed the sequencing experiments. SM performed bioinformatics and statistical analyses. AT, RM, and NJ contributed to manuscript editing. NJ as native English speaker edited the manuscript for language. MK contributed to manuscript editing and figure generation. All authors discussed the results and approved the final submitted version.

### Conflict of Interest Statement

The authors declare that the research was conducted in the absence of any commercial or financial relationships that could be construed as a potential conflict of interest.

## Supplementary Material

Supplementary Figure 1 A260/A280 ratios **(A)** and Genomic Quality Scores **(B)** across different samples and protocols.

Supplementary Figure 2 Representative image of genome quality score.

Supplementary Figure 3 **(A)** Sequence counts of four extraction methods as mentioned in Methods. Horizontal lines indicate the mean of three biological replicates. **(B)** Counts of OTUs observed in of four different DNA extraction methods as mentioned in Methods.
