# Supplementary material for: Evaluation of Methods for the Extraction of Microbial DNA From Vaginal Swabs Used for Microbiome Studies
Source: Front Cell Infect Microbiol. 2019 Jun 6;9:197. doi: 10.3389/fcimb.2019.00197 (PMC6563847; doi:10.3389/fcimb.2019.00197)
Supplement: Supplementary Figure 1 — A260/A280 ratios (A) and Genomic Quality Scores (B) across different samples and protocols. [file Presentation_2.PPTX]

## Slide 1
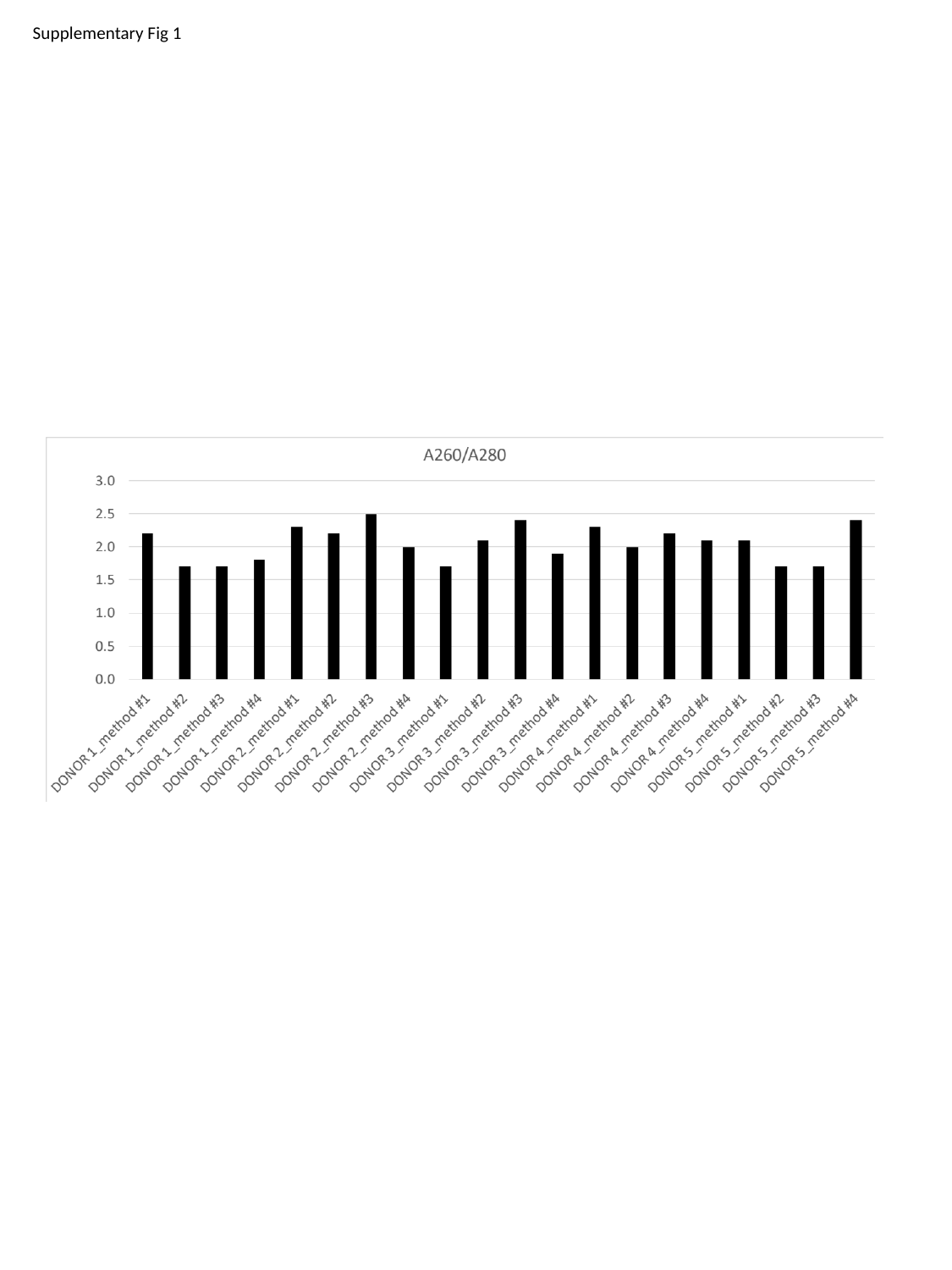

Supplementary Fig 1

## Slide 2
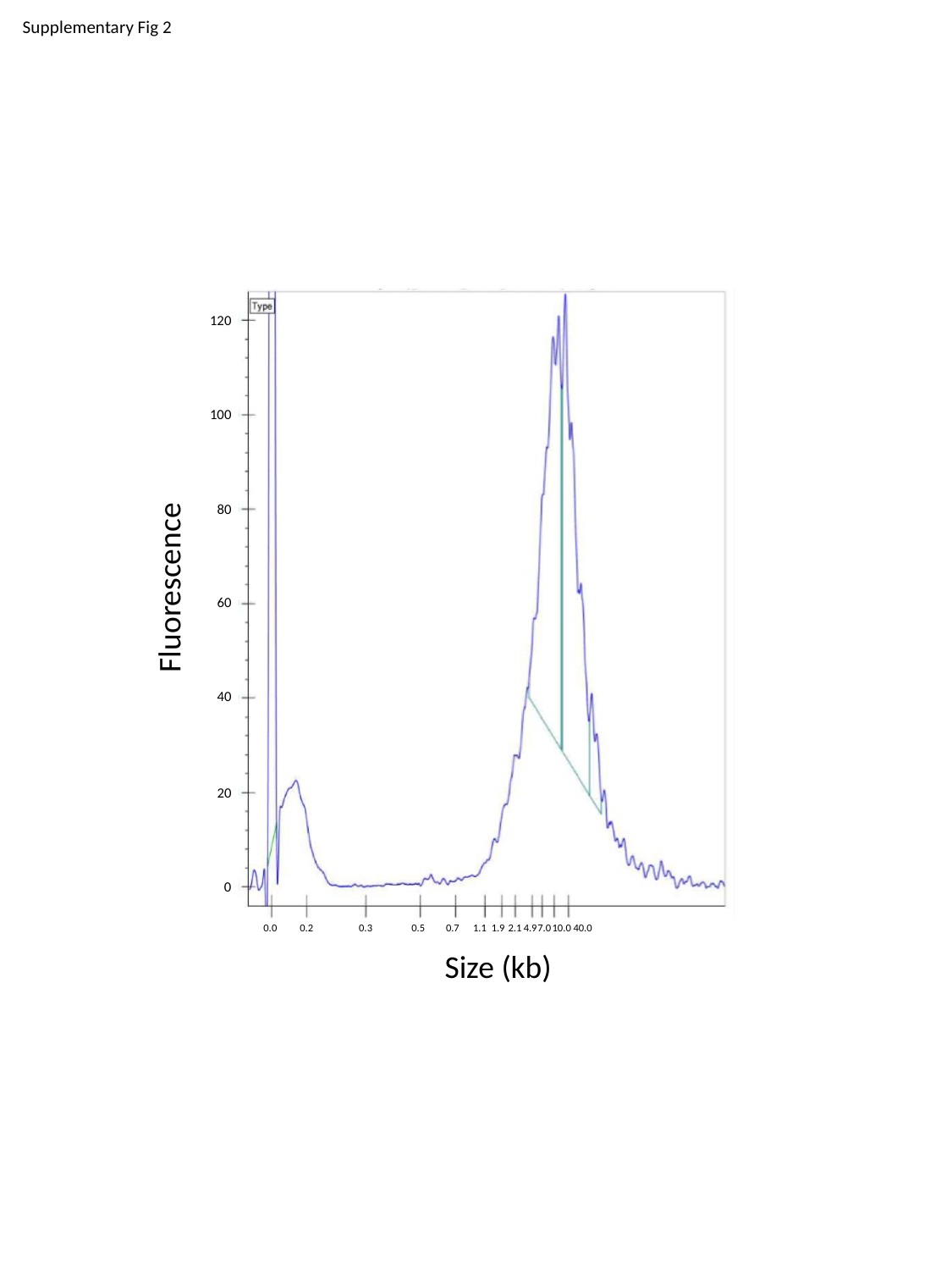

Supplementary Fig 2
80
60
40
20
0
0.0
0.2
0.3
0.5
0.7
1.1
1.9
2.1
4.9
7.0
10.0
40.0
120
100
Fluorescence
Size (kb)

## Slide 3
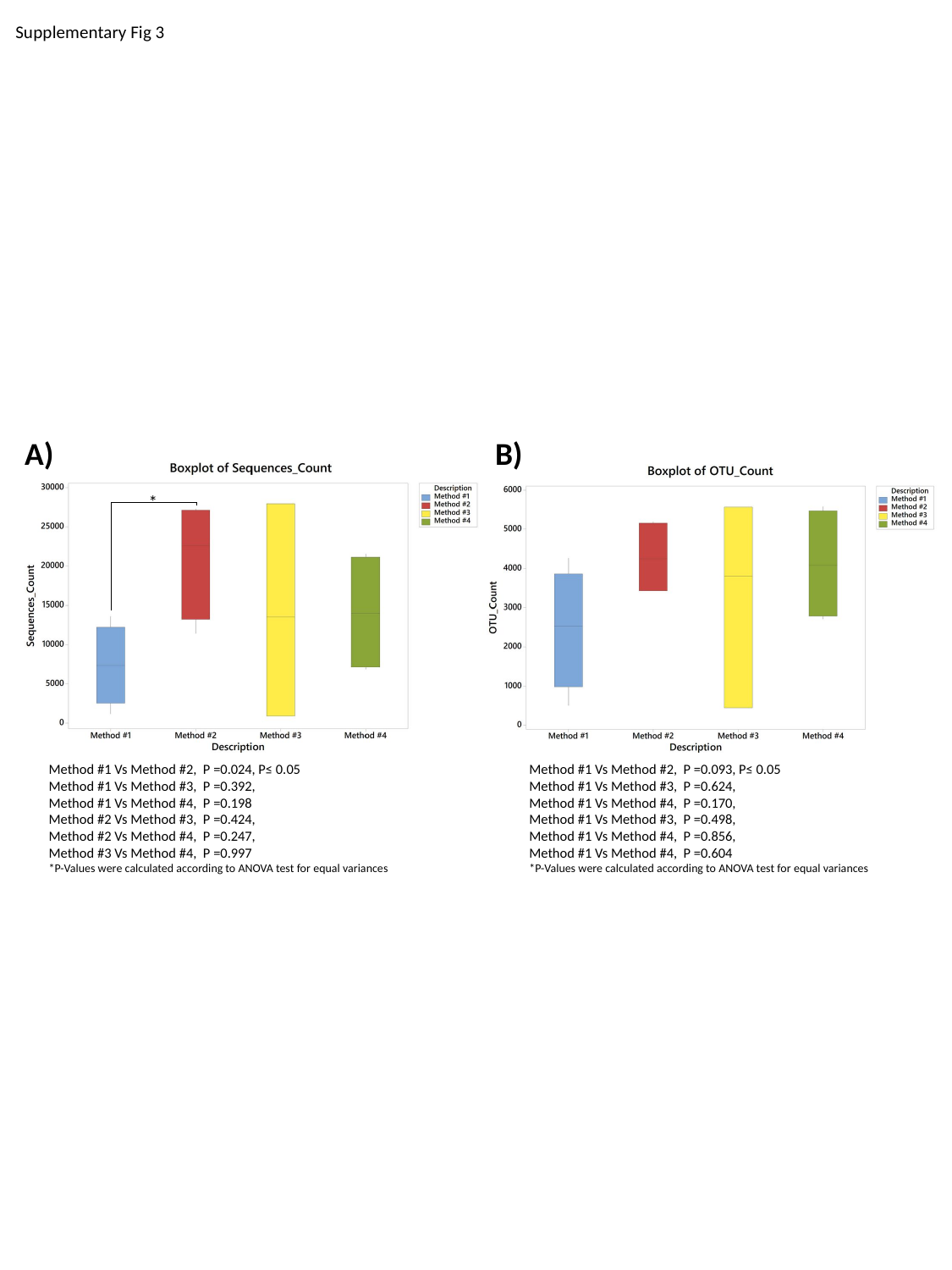

Supplementary Fig 3
A)
B)
*
Method #1 Vs Method #2, P =0.024, P≤ 0.05
Method #1 Vs Method #3, P =0.392,
Method #1 Vs Method #4, P =0.198
Method #2 Vs Method #3, P =0.424,
Method #2 Vs Method #4, P =0.247,
Method #3 Vs Method #4, P =0.997
*P-Values were calculated according to ANOVA test for equal variances
Method #1 Vs Method #2, P =0.093, P≤ 0.05
Method #1 Vs Method #3, P =0.624,
Method #1 Vs Method #4, P =0.170,
Method #1 Vs Method #3, P =0.498,
Method #1 Vs Method #4, P =0.856,
Method #1 Vs Method #4, P =0.604
*P-Values were calculated according to ANOVA test for equal variances
